# Supplementary material for: Hybrid Models and Biological Model Reduction with PyDSTool
Source: PLoS Comput Biol. 2012 Aug 9;8(8):e1002628. doi: 10.1371/journal.pcbi.1002628 (PMC3415397; doi:10.1371/journal.pcbi.1002628)
Supplement: Text S4 — Complete source code for the PyDSTool package (version 0.88.120504). Includes API documentation and help files linking to web pages. This file is identical to the current public release on Sourceforge.net. (ZIP) [file pcbi.1002628.s004.zip › PyDSTool/html/PyDSTool.PyCont.ContClass'-pysrc.html]

xml version="1.0" encoding="ascii"?


PyDSTool.PyCont.ContClass'


| Home | Trees | Indices | Help | PyDSTool |
| --- | --- | --- | --- | --- |

Package PyDSTool ::
Package PyCont ::
Module ContClass'

[hide private]

[frames] | no frames]

# Source Code for Module PyDSTool.PyCont.ContClass'

```
  1  """ ContClass stores continuation curves for a specified model.
 
  2  
 
  3      Drew LaMar, March 2006
 
  4  """ 
  5  
 
  6  from Continuation import EquilibriumCurve, FoldCurve, HopfCurveOne, HopfCurveTwo, \
 
  7                           FixedPointCurve, LimitCycleCurve, UserDefinedCurve 
  8  from misc import * 
  9  from Plotting import pargs, initializeDisplay 
 10  
 
 11  from PyDSTool.Model import Model, findTrajInitiator 
 12  from PyDSTool.Generator import Generator 
 13  from PyDSTool.ModelConstructor import embed 
 14  from PyDSTool import Point, Pointset 
 15  from PyDSTool.common import pickle, Utility, args, filteredDict 
 16  from PyDSTool.utils import remain 
 17  import PyDSTool.Redirector as redirc 
 18  from PyDSTool.errors import * 
 19  try: 
 20      from pylab import * 
 21  except ImportError: 
 22      print "Warning: matplotlib failed to import properly and so is not" 
 23      print "  providing a graphing interface" 
 24  
 
 25  from numpy import dot as matrixmultiply 
 26  from numpy import array, float, complex, int, float64, complex64, int32, \
 
 27      zeros, divide, subtract, Inf, NaN, isfinite, r_, c_, sign, mod, mat, \
 
 28      subtract, divide, transpose, eye, real, imag, all, ndarray 
 29  from numpy import get_numarray_include, get_include 
 30  
 
 31  from PyDSTool.parseUtils import addArgToCalls, wrapArgInCall 
 32  from numpy.distutils.core import setup, Extension 
 33  from distutils.sysconfig import get_python_inc 
 34  import scipy 
 35  import scipy.io as io 
 36  import os, platform, shutil, sys 
 37  from PyDSTool import __path__ as _pydstool_path 
 38  
 
 39  #####
 
 40  _pydstool_path = _pydstool_path[0] 
 41  
 
 42  _classes = ['ContClass'] 
 43  
 
 44  _constants = ['curve_list', 'curve_args_list', 'auto_list'] 
 45  
 
 46  __all__ = _classes + _constants 
 47  #####
 
 48  
 
 49  rout = redirc.Redirector(redirc.STDOUT) 
 50  rerr = redirc.Redirector(redirc.STDERR) 
 51  
 
 52  curve_list = {'EP-C': EquilibriumCurve, 'LP-C': FoldCurve,
 
 53                'H-C1': HopfCurveOne, 'H-C2': HopfCurveTwo,
 
 54                'FP-C': FixedPointCurve, 'LC-C': LimitCycleCurve,
 
 55                'UD-C': UserDefinedCurve} 
 56  
 
 57  curve_args_list = ['verbosity'] 
 58  
 
 59  auto_list = ['LC-C'] 
 60  
 
 61  
 
 62  
 


63 -def distutil_destination():


64      """Makes the goofy destination directory string so that we can find where the distutils
 
 65      fortran compiler puts things.""" 
 66      osname = str.lower(platform.system()) 
 67      pyname = platform.python_version_tuple() 
 68      machinename = platform.machine() 
 69      if osname == 'linux': 
 70          destdir = 'src.'+osname+'-'+machinename+'-'+pyname[0] + '.' + pyname[1] 
 71      elif osname == 'darwin': 
 72          osver = platform.mac_ver()[0].split('.') 
 73          if int(scipy.__version__.split('.')[1]) > 5 and len(osver)>1 and osver != ['']: 
 74              destdir = 'src.macosx-'+osver[0]+'.'+osver[1]+'-'+machinename+'-'+pyname[0] + '.' + pyname[1] 
 75          else: 
 76              destdir = 'src.'+osname+'-'+platform.release()+'-'+machinename+'-'+pyname[0] + '.' + pyname[1] 
 77      elif osname == 'windows': 
 78          destdir = 'src.win32-'+pyname[0]+'.'+pyname[1] 
 79      else: 
 80          destdir = '' 
 81  
 
 82      return destdir

 83  
 
 84  
 


85 -class ContClass(Utility):


86      """Stores continuation curves for a specified model.""" 
 87  
 
 88      curve_list = curve_list 
 89      curve_args_list = curve_args_list 
 90      auto_list = auto_list 
 91  
 


92 -    def __init__(self, model):


93          if isinstance(model, Generator): 
 94              self.model = embed(model, make_copy=False) 
 95              self.gensys = self.model.registry.values()[0] 
 96          else: 
 97              self.model = model 
 98              mi, swRules, globalConRules, nextModelName, reused, \
 
 99                 epochStateMaps, notDone = model._findTrajInitiator(None,
 
100                      0, 0, self.model.icdict, None, None) 
101              self.gensys = mi.model 
102          self._autoMod = None 
103          self.curves = {} 
104          self.plot = pargs()

105  
 


106 -    def __getitem__(self, name):


107          try: 
108              return self.curves[name] 
109          except: 
110              raise KeyError('No curve named ' + str(name))

111  
 


112 -    def __contains__(self, name):


113          return name in self.curves

114  
 


115 -    def __copy__(self):


116          pickledself = pickle.dumps(self) 
117          return pickle.loads(pickledself)

118  
 


119 -    def __deepcopy__(self, memo=None, _nil=[]):


120          pickledself = pickle.dumps(self) 
121          return pickle.loads(pickledself)

122  
 


123 -    def delCurve(self, curvename):


124          try: 
125              del self.curves[curvename] 
126          except KeyError: 
127              raise KeyError("Curve %s does not exist" % curvename)

128  
 


129 -    def newCurve(self, initargs):


130          """Create new curve with arguments specified in the dictionary initargs.""" 
131          curvetype = initargs['type'].upper() 
132  
 
133          if curvetype not in self.curve_list: 
134              raise PyDSTool_TypeError(str(curvetype) + ' not an allowable curve type') 
135  
 
136          # Check name
 
137          cname = initargs['name'] 
138          if 'force' in initargs: 
139              if initargs['force'] and cname in self.curves: 
140                  del self.curves[cname] 
141  
 
142          if cname in self.curves: 
143              raise ValueError('Ambiguous name field: ' + cname \
 
144                             + ' already exists (use force=True to override)') 
145  
 
146          # Check parameters
 
147          if (curvetype != 'UD-C' and self.model.pars == {}) or \
 
148             (curvetype == 'UD-C' and not initargs.has_key('userpars')): 
149              raise ValueError('No parameters defined for this system!') 
150  
 
151          # Process initial point
 
152          initargs = initargs.copy()   # ensures no side-effects outside 
153          if 'initpoint' not in initargs or initargs['initpoint'] is None: 
154              # Default to initial conditions for model
 
155              if self.model.icdict == {}: 
156                  raise ValueError('No initial point defined for this system!') 
157              elif initargs.has_key('uservars'): 
158                  if remain(initargs['uservars'], self.model.icdict.keys()) == []: 
159                      # uservars just used to select a subset of system's regular state vars
 
160                      initargs['initpoint'] = filteredDict(self.model.icdict,
 
161                                                           initargs['uservars']) 
162                  else: 
163                      raise ValueError('No initial point defined for this system!') 
164              else: 
165                  initargs['initpoint'] = self.model.icdict.copy() 
166              #for p in initargs['freepars']:
 
167              #    initargs['initpoint'][p] = self.model.pars[p]
 
168          else: 
169              if isinstance(initargs['initpoint'], dict): 
170                  initargs['initpoint'] = initargs['initpoint'].copy() 
171                  #for p in initargs['freepars']:
 
172                  #    if p not in initargs['initpoint'].keys():
 
173                  #        initargs['initpoint'][p] = self.model.pars[p]
 
174              elif isinstance(initargs['initpoint'], str): 
175                  curvename, pointname = initargs['initpoint'].split(':') 
176                  pointtype = pointname.strip('0123456789') 
177                  if not self.curves.has_key(curvename): 
178                      raise KeyError('No curve of name ' + curvename + ' exists.') 
179                  else: 
180                      point = self.curves[curvename].getSpecialPoint(pointtype, pointname) 
181                      if point is None: 
182                          raise KeyError('No point of name ' + pointname + ' exists.') 
183                      else: 
184                          initargs['initpoint'] = point 
185  
 
186              # Separate from if-else above since 'str' clause returns type Point
 
187              if isinstance(initargs['initpoint'], Point): 
188                  # Check to see if point contains a cycle.  If it does, assume
 
189                  #   we are starting at a cycle and save it in initcycle
 
190                  for v in initargs['initpoint'].labels.itervalues(): 
191                      if v.has_key('cycle'): 
192                          initargs['initcycle'] = v   # Dictionary w/ cycle, name, and tangent information 
193  
 
194                  # Save initial point information
 
195                  initargs['initpoint'] = initargs['initpoint'].copy().todict() 
196                  #for p in initargs['freepars']:
 
197                  #    if p not in initargs['initpoint'].keys():
 
198                  #        initargs['initpoint'][p] = self.model.pars[p]
 
199  
 
200          # Process cycle
 
201          if 'initcycle' in initargs: 
202              if isinstance(initargs['initcycle'], ndarray): 
203                  c0 = {} 
204                  c0['data'] = args(V = {'udotps': None, 'rldot': None}) 
205                  c0['cycle'] = Pointset({'coordnames': self.gensys.funcspec.vars,
 
206                                          'coordarray': initargs['initcycle'][1:,:].copy(),
 
207                                          'indepvarname': 't',
 
208                                          'indepvararray': initargs['initcycle'][0,:].copy()
 
209                                         }) 
210                  initargs['initcycle'] = c0 
211              elif isinstance(initargs['initcycle'], Pointset): 
212                  c0 = {} 
213                  c0['data'] = args(V = {'udotps': None, 'rldot': None}) 
214                  c0['cycle'] = initargs['initcycle'] 
215                  initargs['initcycle'] = c0 
216  
 
217          # Load auto module if required
 
218          automod = None 
219          if curvetype in auto_list: 
220              if self._autoMod is None: 
221                  self.loadAutoMod() 
222              automod = self._autoMod 
223  
 
224          self.curves[cname] = self.curve_list[curvetype](self.model, self.gensys, automod, self.plot, initargs)

225  
 
226      # Export curve data to Matlab file format
 


227 -    def exportMatlab(self, filename=None):


228  
 
229          if not filename: 
230              filename = self.model.name + '.mat' 
231  
 
232          savedict = {} 
233  
 
234          print self.__dict__['curves'].keys() 
235          # Save data for each curve with different prefix
 
236          for name in self.__dict__['curves'].keys(): 
237              if self[name].curvetype not in ['EP-C', 'LC-C']: 
238                  print "Can't save curve type", self[name].curvetype, "yet. (", name, ")" 
239                  continue 
240              # Can save equilibrium curves
 
241              else: 
242                  curve = self[name].sol 
243                  N = len(curve) 
244                  # Currently ignoring the extra labels (B, etx)
 
245                  if self[name].curvetype == 'EP-C': 
246                      label = 'EP' 
247                  elif self[name].curvetype == 'LC-C': 
248                      label = 'LC' 
249  
 
250  
 
251                  # Add variables
 
252                  for key in curve.coordnames: 
253                      savedict[name+'_'+key] = array(curve[key]) 
254  
 
255                  # Add stabilities
 
256                  if 'stab' in curve[0].labels[label].keys(): 
257                      savedict[name+'_stab'] = [curve[x].labels[label]['stab'] for x in range(N)] 
258                      temp = zeros(N) 
259                      for x in range(N): 
260                          if savedict[name+'_stab'][x] == 'S': 
261                              temp[x] = -1 
262                          elif savedict[name+'_stab'][x] == 'U': 
263                              temp[x] = 1 
264                      savedict[name+'_stab'] = array(temp) 
265  
 
266                  # Add domain
 
267                  if 'domain' in curve[0].labels[label].keys(): 
268                      savedict[name+'_domain'] = [curve[x].labels[label]['domain'] for x in range(N)] 
269                      temp = zeros(N) 
270                      for x in range(N): 
271                          if savedict[name+'_domain'][x] == 'inside': 
272                              temp[x] = -1 
273                          elif savedict[name+'_domain'][x] == 'outside': 
274                              temp[x] = 1 
275                      savedict[name+'_domain'] = array(temp) 
276  
 
277                  # Add data
 
278                  if 'data' in curve[0].labels[label].keys(): 
279                      # Add eigenvalues
 
280                      if 'evals' in curve[0].labels[label]['data'].keys(): 
281                          dim = len(curve[0].labels[label]['data']['evals']) 
282                          evltmp = [[] for x in range(dim)] 
283                          for x in range(dim): 
284                              evltmp[x] = array([curve[y].labels[label]['data']['evals'][x] for y in range(N)]) 
285                          savedict[name+'_evals'] = array(evltmp) 
286  
 
287                      # Add ds
 
288                      if 'ds' in curve[0].labels[label]['data'].keys(): 
289                          savedict[name+'_ds'] = array([curve[y].labels[label]['data']['ds'] for y in range(N)]) 
290  
 
291                      # Add eigenvectors
 
292                      if 'evecs' in curve[0].labels[label]['data'].keys(): 
293                          #dim = len(curve.coordnames) - 1
 
294                          dim = len(curve[0].labels[label]['data']['evecs'][0]) 
295                          evectmp = [] 
296                          for x in range(dim): 
297                              for y in range(dim): 
298                                  evectmp.append(array([curve[z].labels[label]['data']['evecs'][x][y] for z in range(N)])) 
299                          savedict[name+'_evecs'] = array(evectmp) 
300  
 
301                      # Add V
 
302                      if 'V' in curve[0].labels[label]['data'].keys(): 
303                          for key in curve[0].labels[label]['data']['V'].keys(): 
304                              savedict[name+'_V_'+key] = array([curve[x].labels[label]['data']['V'][key] for x in range(N)]) 
305  
 
306          # Save the dictionary in matlab format
 
307          io.savemat(filename, savedict)

308  
 
309  
 


310 -    def exportGeomview(self, coords=None, filename="geom.dat"):


311          if  coords is not None and len(coords) == 3: 
312              GeomviewOutput = "(progn (geometry " + self.model.name + " { LIST {: axes_" + self.model.name + "}" 
313              for cname, curve in self.curves.iteritems(): 
314                  GeomviewOutput += " {: " + cname + "}" 
315              GeomviewOutput += "}))\n\n" 
316  
 
317              # Get axes limits
 
318              alim = [[Inf,-Inf],[Inf,-Inf],[Inf,-Inf]] 
319              for cname, curve in self.curves.iteritems(): 
320                  for n in range(len(coords)): 
321                      alim[n][0] = min(alim[n][0], min(curve.sol[coords[n]].toarray())) 
322                      alim[n][1] = max(alim[n][1], max(curve.sol[coords[n]].toarray())) 
323  
 
324              GeomviewOutput += "(progn (hdefine geometry axes_" + self.model.name + " { appearance { linewidth 2 } SKEL 4 3 " + \
 
325                  "0 0 0 1 0 0 0 1 0 0 0 1 " + \
 
326                  "2 0 1 1 0 0 1 2 0 2 0 1 0 1 2 0 3 0 0 1 1})\n\n" 
327  
 
328              for cname, curve in self.curves.iteritems(): 
329                  GeomviewOutput += "(hdefine geometry " + cname + " { LIST {: curve_" + cname + "} {: specpts_" + cname + "}})\n\n" 
330  
 
331                  GeomviewOutput += "(hdefine geometry curve_" + cname + " { appearance { linewidth 2 } SKEL " + \
 
332                      repr(len(curve.sol)) + " " + repr(len(curve.sol)-1) 
333                  for n in range(len(curve.sol)): 
334                      GeomviewOutput += " " + repr((curve.sol[n][coords[0]]-alim[0][0])/(alim[0][1]-alim[0][0])) + \
 
335                                             " " + repr((curve.sol[n][coords[1]]-alim[1][0])/(alim[1][1]-alim[1][0])) + \
 
336                                             " " + repr((curve.sol[n][coords[2]]-alim[2][0])/(alim[2][1]-alim[2][0])) 
337                  for n in range(len(curve.sol)-1): 
338                      GeomviewOutput += " 2 " + repr(n) + " " + repr(n+1) + " 0 0 0 1" 
339  
 
340                  GeomviewOutput += "})\n\n" 
341  
 
342              GeomviewOutput += ")\n" 
343  
 
344              f = open(filename, "w") 
345              f.write(GeomviewOutput) 
346              f.close() 
347          else: 
348              raise Warning("Coordinates not specified or not of correct dimension.")

349  
 


350 -    def display(self, coords=None, curves=None, figure=None, axes=None, stability=False, domain=False, **plot_args):


351          """Plot all curves in coordinates specified by coords.
 
352  
 
353             Inputs:
 
354  
 
355                 coords -- tuple of coordinates (None defaults to the first free
 
356                     parameter and the first state variable)
 
357          """ 
358          if coords is not None and len(coords) == 3: 
359              self.exportGeomview(coords) 
360              return 
361  
 
362          if curves is None: 
363              curves = self.curves.keys() 
364  
 
365          plot_curves = [] 
366          for curve in curves: 
367              if self.curves.has_key(curve): 
368                  plot_curves.append(curve) 
369              else: 
370                  print "Warning: Curve " + curve + " does not exist." 
371  
 
372          if len(plot_curves) > 0: 
373              initializeDisplay(self.plot, figure=figure, axes=axes) 
374  
 
375          for curve in plot_curves: 
376              self.curves[curve].display(coords, figure=figure, axes=axes, stability=stability, domain=domain, init_display=False, **plot_args)

377  
 


378 -    def computeEigen(self):


379          for curve in self.curves.itervalues(): 
380              curve.computeEigen()

381  
 


382 -    def info(self):


383          print self.__repr__() 
384          #print "  Variables : %s"%', '.join(self.model.allvars)
 
385          #print "  Parameters: %s\n"%', '.join(self.model.pars.keys())
 
386          print "Containing curves: " 
387          for c in self.curves: 
388              print "  " + c + " (type " + self.curves[c].curvetype + ")"

389  
 


390 -    def update(self, args):


391          """Update parameters for all curves.""" 
392          for c in args.keys(): 
393              if c not in curve_args_list: 
394                  args.pop(c) 
395  
 
396          for v in self.curves.values(): 
397              v.update(args)

398  
 


399 -    def loadAutoMod(self):


400          thisplatform = platform.system() 
401          if thisplatform == 'Windows': 
402              self._dllext = ".pyd" 
403          elif thisplatform in ['Linux', 'IRIX', 'Solaris', 'SunOS', 'Darwin']: 
404              self._dllext = '.so' 
405          else: 
406              print "Shared library extension not tested on this platform." 
407              print "If this process fails please report the errors to the" 
408              print "developers." 
409              self._dllext = '.so' 
410  
 
411          self._compilation_tempdir = os.path.join(os.getcwd(),
 
412                                                        "auto_temp") 
413          if not os.path.isdir(self._compilation_tempdir): 
414              try: 
415                  assert not os.path.isfile(self._compilation_tempdir), \
 
416                       "A file already exists with the same name" 
417                  os.mkdir(self._compilation_tempdir) 
418              except: 
419                  print "Could not create compilation temp directory " + \
 
420                        self._compilation_tempdir 
421                  raise 
422          self._compilation_sourcedir = os.path.join(_pydstool_path,"PyCont/auto/module") 
423          self._vf_file = self.gensys.name+"_vf.c" 
424          self._vf_filename_ext = "_"+self._vf_file[:-2] 
425          if not (os.path.isfile(os.path.join(os.getcwd(),
 
426                                  "auto"+self._vf_filename_ext+".py")) and \
 
427                  os.path.isfile(os.path.join(os.getcwd(),
 
428                                  "_auto"+self._vf_filename_ext+self._dllext))): 
429              if True: 
430                  self.funcspec = self.gensys.funcspec.recreate('c') 
431                  self.makeAutoLibSource() 
432                  self.compileAutoLib() 
433              else: 
434                  print "Build the library using the makeLib method, or in " 
435                  print "stages using the makeLibSource and compileLib methods." 
436  
 
437          try: 
438              self._autoMod = __import__("auto"+self._vf_filename_ext, globals()) 
439          except: 
440              print "Error loading auto module." 
441              raise

442  
 


443 -    def forceAutoLibRefresh(self):


444          """forceAutoLibRefresh should be called after event contents are changed,
 
445          or alterations are made to the right-hand side of the ODEs.
 
446  
 
447          Currently this function does NOT work!""" 
448  
 
449          # (try to) free auto module from namespace
 
450          delfiles = True 
451          try: 
452              del(sys.modules["_auto"+self._vf_filename_ext]) 
453              del(sys.modules["auto"+self._vf_filename_ext]) 
454          except NameError: 
455              # modules weren't loaded, so nothing to do
 
456              delfiles = False 
457          if delfiles: 
458              gc.collect() 
459              # still not able to delete these files!!!!! Argh!
 
460          print "Cannot rebuild library without restarting session. Sorry." 
461          print "Try asking the Python developers to make a working module" 
462          print "unimport function!"

463  
 


464 -    def makeAutoLib(self, libsources=[], libdirs=[], include=[]):


465          """makeAutoLib calls makeAutoLibSource and then the compileAutoLib method.
 
466          To postpone compilation of the source to a DLL, call makeAutoLibSource()
 
467          separately.""" 
468          self.makeAutoLibSource(include) 
469          self.compileAutoLib(libsources, libdirs)

470  
 


471 -    def makeAutoLibSource(self, include=[]):


472          """makeAutoLibSource generates the C source for the vector field specification.
 
473          It should be called only once per vector field.""" 
474  
 
475          # Make vector field (and event) file for compilation
 
476          assert isinstance(include, list), "includes must be in form of a list" 
477          # codes for library types (default is USERLIB, since compiler will look in standard library d
 
478          STDLIB = 0 
479          USERLIB = 1 
480          libinclude = dict([('math.h', STDLIB), ('stdio.h', STDLIB), ('stdlib.h', STDLIB),
 
481                        ('string.h', STDLIB), ('autovfield.h', USERLIB),
 
482                        ('auto_c.h', USERLIB)]) 
483          include_str = '#include "auto_f2c.h"\n' # This must come first 
484          for libstr, libtype in libinclude.iteritems(): 
485              if libtype == STDLIB: 
486                  quoteleft = '<' 
487                  quoteright = '>' 
488              else: 
489                  quoteleft = '"' 
490                  quoteright = '"' 
491              include_str += "#include " + quoteleft + libstr + quoteright + "\n" 
492          if include != []: 
493              assert isUniqueSeq(include), "list of library includes must not contain repeats" 
494              for libstr in include: 
495                  if libstr in libinclude: 
496                      # don't repeat libraries
 
497                      print "Warning: library '" + libstr + "' already appears in list"\
 
498                            + " of imported libraries" 
499                  else: 
500                      include_str += "#include " + '"' + libstr + '"\n' 
501  
 
502          # f2c auto conventions (dirty trick!)
 
503          #define_str = "\n#define double    doublereal\n#define int   integer\n\n"
 
504          define_str = "" 
505  
 
506          allfilestr = "/*  Vector field and other functions for Auto continuer.\n " \
 
507              + "  This code was automatically generated by PyDSTool, but may be modified " \
 
508              + "by hand. */\n\n" + include_str + define_str + """
 
509  double *gICs;
 
510  double **gBds;
 
511  double globalt0;
 
512  
 
513  static double pi = 3.1415926535897931;
 
514  
 
515  """ 
516          pardefines = "" 
517  ##        parundefines = ""
 
518          vardefines = "" 
519  ##        varundefines = ""
 
520          inpdefines = "" 
521  ##        inpundefines = ""
 
522          # sorted version of var, par, and input names
 
523          vnames = self.gensys._var_ixmap 
524          pnames = self.funcspec.pars 
525          inames = self.funcspec.inputs 
526          pnames.sort() 
527          inames.sort() 
528          for i in xrange(self.gensys.numpars): 
529              p = pnames[i] 
530              # add to defines (WATCH OUT FOR PERIOD _T!!!)
 
531              if (i < 10): 
532                  pardefines += self.funcspec._defstr+" "+p+"\tp_["+str(i)+"]\n" 
533              elif (i >= 10): 
534                  pardefines += self.funcspec._defstr+" "+p+"\tp_["+str(i+40)+"]\n" 
535          # add period _T
 
536          pardefines += self.funcspec._defstr+" _T\tp_[10]\n" 
537          for i in xrange(self.gensys.dimension): 
538              v = vnames[i] 
539              # add to defines
 
540              vardefines += self.funcspec._defstr+" "+v+"\tY_["+str(i)+"]\n" 
541  ##            # add to undefines
 
542  ##            varundefines += self.funcspec._undefstr+" "+v+"\n"
 
543          for i in xrange(len(self.funcspec.inputs)): 
544              inp = inames[i] 
545              # add to defines
 
546              inpdefines += self.funcspec._defstr+" "+inp+"\txv_["+str(i)+"]\n" 
547  ##            # add to undefines
 
548  ##            inpundefines += self.funcspec._undefstr+" "+inp+"\n"
 
549          allfilestr += "\n/* Variable, parameter, and input definitions: */ \n" \
 
550                        + pardefines + vardefines + inpdefines + "\n" 
551          # add signature for auxiliary functions
 
552          if self.funcspec.auxfns: 
553              allfilestr += "\n" 
554              for finfo in self.funcspec.auxfns.values(): 
555                  allfilestr += finfo[1] + ";\n" 
556          allfilestr += "\nvoid auxvars(unsigned, unsigned, double, double*, double*, " \
 
557                + "double*, unsigned, double*, unsigned, double*);\n" \
 
558                + """void jacobian(unsigned, unsigned, double, double*, double*, double**, unsigned, double*, unsigned, double*);
 
559  void jacobianParam(unsigned, unsigned, double, double*, double*, double**, unsigned, double*, unsigned, double*);
 
560  """ 
561          if self.funcspec.auxvars == []: 
562              allfilestr += "int N_AUXVARS = 0;\n\n\n" 
563          else: 
564              allfilestr += "int N_AUXVARS = " + str(len(self.funcspec.auxvars)) \
 
565                         + ";\n\n\n" 
566          allfilestr += self.funcspec.spec[0] + "\n\n" 
567  
 
568          if self.funcspec.auxfns: 
569              for fname, finfo in self.funcspec.auxfns.iteritems(): 
570                  fbody = finfo[0] 
571                  # subs _p into auxfn-to-auxfn calls (but not to the signature)
 
572                  fbody_parsed = addArgToCalls(fbody,
 
573                                          self.funcspec.auxfns.keys(),
 
574                                          "p_, wk_, xv_", notFirst=fname) 
575                  if 'initcond' in self.funcspec.auxfns: 
576                      # convert 'initcond(x)' to 'initcond("x")' for
 
577                      # compatibility with C syntax, but don't affect the
 
578                      # function signature!
 
579                      fbody_parsed = wrapArgInCall(fbody_parsed,
 
580                                          'initcond', '"', notFirst=True) 
581                  allfilestr += "\n" + fbody_parsed + "\n\n" 
582          # add auxiliary variables (shell of the function always present)
 
583          # add event functions
 
584          allfilestr += self.funcspec.auxspec[0] 
585          # if jacobians or mass matrix not present, fill in dummy
 
586          if not self.gensys.haveJacobian(): 
587              allfilestr += """
 
588  void jacobian(unsigned n_, unsigned np_, double t, double *Y_, double *p_, double **f_, unsigned wkn_, double *wk_, unsigned xvn_, double *xv_) {
 
589  }
 
590  """ 
591          if not self.gensys.haveJacobian_pars(): 
592              allfilestr += """
 
593  void jacobianParam(unsigned n_, unsigned np_, double t, double *Y_, double *p_, double **f_, unsigned wkn_, double *wk_, unsigned xvn_, double *xv_) {
 
594  }
 
595  """ #+ "\n/* Variable and parameter substitutions undefined:*/\n" + parundefines + varundefines + "\n" 
596          # write out C file
 
597          vffile = os.path.join(self._compilation_tempdir, self._vf_file) 
598          try: 
599              file = open(vffile, 'w') 
600              #allfilestr = allfilestr.replace("double ","doublereal ")
 
601              #allfilestr = allfilestr.replace("double*","doublereal *")
 
602              #allfilestr = allfilestr.replace("double**","doublereal **")
 
603              file.write(allfilestr) 
604              file.close() 
605          except IOError, e: 
606              print "Error opening file "+self._vf_file+" for writing" 
607              raise IOError, e

608  
 


609 -    def compileAutoLib(self, libsources=[], libdirs=[]):


610          """compileAutoLib generates a python extension DLL with continuer and vector
 
611          field compiled and linked.
 
612  
 
613          libsources list allows additional library sources to be linked.
 
614          libdirs list allows additional directories to be searched for
 
615            precompiled libraries.""" 
616  
 
617          if os.path.isfile(os.path.join(os.getcwd(),
 
618                                  "_auto"+self._vf_filename_ext+self._dllext)): 
619              # then DLL file already exists and we can't overwrite it at this
 
620              # time
 
621              proceed = False 
622              print "\n" 
623              print "-----------------------------------------------------------" 
624              print "Present limitation of Python: Cannot rebuild library" 
625              print "without exiting Python and deleting the shared library" 
626              print "   " + str(os.path.join(os.getcwd(),
 
627                                  "_auto"+self._vf_filename_ext+self._dllext)) 
628              print "by hand! If you made any changes to the system you should" 
629              print "not proceed with running the integrator until you quit" 
630              print "and rebuild." 
631              print "-----------------------------------------------------------" 
632              print "\n" 
633          else: 
634              proceed = True 
635          if not proceed: 
636              print "Did not compile shared library." 
637              return 
638          if self._autoMod is not None: 
639              self.forceAutoLibRefresh() 
640          vffile = os.path.join(self._compilation_tempdir, self._vf_file) 
641          try: 
642              ifacefile_orig = open(os.path.join(self._compilation_sourcedir,
 
643                                                 "automod.i"), 'r') 
644              ifacefile_copy = open(os.path.join(self._compilation_tempdir,
 
645                                         "auto_"+self._vf_file[:-2]+".i"), 'w') 
646              firstline = ifacefile_orig.readline() 
647              ifacefile_copy.write('%module auto_'+self._vf_file[:-2]+'\n') 
648              iffilestr = ifacefile_orig.read() 
649              ifacefile_copy.write(iffilestr) 
650              ifacefile_orig.close() 
651              ifacefile_copy.close() 
652          except IOError: 
653              print "automod.i copying error in auto compilation directory" 
654              raise 
655  
 
656          swigfile = os.path.join(self._compilation_tempdir,
 
657                                  "auto"+self._vf_filename_ext+".i") 
658          automodfile = os.path.join(self._compilation_sourcedir, "automod.c") 
659          interfacefile = os.path.join(self._compilation_sourcedir, "interface.c") 
660  
 
661          # source files
 
662          if not (all([os.path.isfile(os.path.join(self._compilation_tempdir,
 
663                             sf)) for sf in ['auto'+self._vf_filename_ext+'_wrap.o',
 
664                                             'auto'+self._vf_filename_ext+'.py',
 
665                                             '_auto'+self._vf_filename_ext+'.def']])): 
666              modfilelist = [swigfile] 
667          else: 
668              modfilelist = [] 
669          # FOR DIST (ADD)
 
670          modfilelist.extend([os.path.join(self._compilation_sourcedir, "../src/"+x) \
 
671              for x in ['auto.c','autlib1.c','autlib2.c','autlib3.c','autlib4.c','autlib5.c', \
 
672              'eispack.c', 'conpar.c','setubv.c','reduce.c','dmatrix.c','fcon.c','libf2c/cabs.c','libf2c/d_lg10.c', \
 
673              'libf2c/i_nint.c','libf2c/pow_di.c','libf2c/r_lg10.c','libf2c/z_exp.c','libf2c/d_imag.c', \
 
674              'libf2c/d_sign.c','libf2c/i_dnnt.c','libf2c/pow_dd.c','libf2c/pow_ii.c','libf2c/z_abs.c', \
 
675              'libf2c/z_log.c']]) 
676  
 
677          modfilelist.extend([automodfile, interfacefile, vffile]) 
678          # FOR DIST (SUBTRACT)
 
679          #modfilelist.extend(libsources)
 
680  
 
681          # script args
 
682          script_args = ['-q', 'build', '--build-lib=.', #+os.getcwd(), # '-t/',
 
683                   '-tauto_temp', #+self._compilation_tempdir,
 
684                   '--build-base=auto_temp'] #+self._compilation_sourcedir] 
685          if self.gensys._compiler != '': 
686              script_args.append('-c'+str(self.gensys._compiler)) 
687  
 
688          # include directories for libraries
 
689          narraydir = get_numarray_include() 
690          npydir = get_include() 
691  
 
692          incdirs = [narraydir, npydir, os.getcwd(), os.path.join(self._compilation_sourcedir,"include"),
 
693              self._compilation_tempdir, os.path.join(_pydstool_path,"PyCont/auto/src/include")] 
694          incdirs.extend(libdirs) 
695  
 
696          # libraries
 
697          # FOR DIST (SUBTRACT)
 
698          #libdirs.append(os.path.join(_pydstool_path, "PyCont/auto/lib"))
 
699          #libsources.append('auto2000')
 
700  
 
701          # Use distutils to perform the compilation of the selected files
 
702          rout.start()    # redirect stdout 
703          try: 
704              distobject = setup(name = "Auto 2000 continuer",
 
705                    author = "PyDSTool (automatically generated)",
 
706                    script_args = script_args,
 
707                    ext_modules = [Extension("_auto"+self._vf_filename_ext,
 
708                                   sources=modfilelist,
 
709                                   include_dirs=incdirs,
 
710                                   extra_compile_args=['-w', '-D__PYTHON__', '-std=c99', '-m32'],
 
711                                   extra_link_args=['-w', '-m32'],
 
712                                   library_dirs=libdirs+['./'],
 
713                                   libraries=libsources)]) 
714          except: 
715              print "\nError occurred in generating Auto system..." 
716              print sys.exc_info()[0], sys.exc_info()[1] 
717              raise RuntimeError 
718          rout.stop()    # restore stdout 
719          try: 
720              # move library files into the user's CWD
 
721              distdestdir = distutil_destination() 
722              if swigfile in modfilelist or not \
 
723                 os.path.isfile(os.path.join(self._compilation_tempdir,
 
724                                  "auto"+self._vf_filename_ext+".py")): 
725                  shutil.move(os.path.join(os.getcwd(),
 
726                                  self._compilation_tempdir, distdestdir,
 
727                                  "auto_temp",
 
728                                   "auto"+self._vf_filename_ext+".py"),
 
729                              os.path.join(os.getcwd(),
 
730                                   "auto"+self._vf_filename_ext+".py")) 
731          except: 
732              print "\nError occurred in generating Auto system" 
733              print "(while moving library extension modules to CWD)" 
734              print sys.exc_info()[0], sys.exc_info()[1] 
735              raise RuntimeError

736  
 


737 -    def __repr__(self):


738          return 'ContClass of model %s'%self.model.name

739  
 
740      __str__ = __repr__

741
```

  


| Home | Trees | Indices | Help | PyDSTool |
| --- | --- | --- | --- | --- |

|  |  |
| --- | --- |
| Generated by Epydoc 3.0.1 on Fri May 4 15:24:16 2012 | http://epydoc.sourceforge.net |
